# Supplementary material for: Implementation of a care-pathway at the emergency department for older people presenting with nonspecific complaints; a protocol for a multicenter parallel cohort study
Source: PLoS One. 2023 Aug 29;18(8):e0290733. doi: 10.1371/journal.pone.0290733 (PMC10464958; doi:10.1371/journal.pone.0290733)
Supplement: S1 File — (DOCX) [file pone.0290733.s001.docx]

**S1. PRM Acute Care**

## **Questionnaire: the perceived quality of care in the Emergengy Department**

You are treated in the Emergency Department. We would like to know how you perceived the delivered care and if you feel you are treated well in our Emergency Department.

Could you please recall your Emergency Department visit and answer the following questions?

|  | | |  | **No complaints Very severe complaints** | | | | | |
| --- | --- | --- | --- | --- | --- | --- | --- | --- | --- |
| 1. What was the severity of your complaints on arrival at the Emergency Department? | | |  | 0 1 2 3 4 5 6 7 8 9 10 | | | | | |
| 1. What was the severity of your complaints on departure from the Emergency Department? 2. Have your complaints been treated? | | |  | 0 1 2 3 4 5 6 7 8 9 10   - Yes, and the treatment has helped - Yes, but the treatment did not help enough - Yes, and I am still treated - No, but I would have liked to have a treatment - No, but I did not want treatment | | | | | |
|  |  | **Not at all** | | | **Barely** | **Moderate** | **Fairly** | **Good** | **Completely** |
| 1. Did you understand the explanation in the Emergency Department about the cause of your complaints? |  | 1 | | | 2 | 3 | 4 | 5 | 6 |
|  |  | *I did not get an explanation about the cause of my complaints* | | | | | | | |
|  |  |  | | |  |  |  |  |  |
| 1. Do you understand why additional diagnostics and treatments were executed in the Emergency Department? |  | 1 | | | 2 | 3 | 4 | 5 | 6 |
| 1. Do you understand the next steps in the treatment of your condition, during admission or at home? |  | 1 | | | 2 | 3 | 4 | 5 | 6 |
|  |  | **Not at all** | | | **Barely** | **Moderate** | **Fairly** | **Good** | **Completely** |
| 1. Did you feel reassured after your visit of the Emergency Department? |  | 1 | | | 2 | 3 | 4 | 5 | 6 |
| 1. Do you think you/your complaints needed further examination? |  | 1 | | | 2 | 3 | 4 | 5 | 6 |
| 1. Are you satisfied with the total length of stay in the Emergency Department? |  | 1 | | | 2 | 3 | 4 | 5 | 6 |
|  |  |  | | |  |  |  |  |  |
| 1. Did you feel safe during your visit in the Emergency Department? |  | 1 | | | 2 | 3 | 4 | 5 | 6 |
| 1. Did the healthcare professionals listen attentively to you, during your stay in the Emergency Department? |  | 1 | | | 2 | 3 | 4 | 5 | 6 |
| 1. Did you have trust in the expertise of the healthcare professionals in the Emergency Department?   ***Additional questions*** |  | 1 | | | 2 | 3 | 4 | 5 | 6 |
|  |  | **Very poor Very good** | | | | | | | |
| 1. How would you grade the Emergency Department in general? (on a scale form zero tot ten) |  | 0 1 2 3 4 5 6 7 8 9 10 | | | | | | | |
