# Supplementary material for: Implementation of a care-pathway at the emergency department for older people presenting with nonspecific complaints; a protocol for a multicenter parallel cohort study
Source: PLoS One. 2023 Aug 29;18(8):e0290733. doi: 10.1371/journal.pone.0290733 (PMC10464958; doi:10.1371/journal.pone.0290733)
Supplement: S3 File — (PDF) [file pone.0290733.s003.pdf]

Care pathway for older adults presenting with non-specific complaints at the emergency department

(October 2020)

PROTOCOL TITLE 'Care pathway for older adults presenting with non-specific complaints at the emergency department'

**Protocol ID**	Care pathway for older adults presenting with non-specific complaints at the emergency department
**Short title**	Care pathway for elderly with NSCs at the ED
**EudraCT number**	
**Version**	7.0
**Date**	29-12-2020
**Coordinating investigator/project leader**	Prof. dr. H.R. Haak, internist acute medicine<br>Department of Internal Medicine<br>Maxima Medisch Centrum<br>De Run 4600, 5504 DB Veldhoven<br>e-mail: h.haak@mmc.nl
**Participants**	Drs. S.L.E. Lambooij<br>Department of Internal Medicine<br>Maxima Medisch Centrum<br>De Run 4600, 5504 DB Veldhoven<br>e-mail: e.lambooij@mmc.nl<br><br>Drs. M.N.T. Kremers, MSc<br>Department of Internal Medicine<br>Maxima Medisch Centrum<br>De Run 4600, 5504 DB Veldhoven<br>Email: marjolein.kremers@mmc.nl<br><br>Drs. M.G.A.M. van der Velde, MSc<br>Department of Internal Medicine<br>Maxima Medisch Centrum<br>De Run 4600, 5504 DB Veldhoven<br>e-mail: Marleen.van.der.Velde@mmc.nl<br><br>Drs. R.W. Vingerhoets, klinisch geriater<br>Elisabeth TweeSteden Ziekenhuis<br>Hilvarenbeekseweg 60 5022 GC Tilburg<br>e-mail: r.vingerhoets@etz.nl<br><br>Drs. P. Wouda, kaderhuisarts ouderengeneeskunde<br>Huisartsencentrum Parklaan-Wouda<br>Parklaan 12, 5613 BE Eindhoven<br>e-mail: P.Wouda@zorggroepdoh.nl<br><br>C. de Vries, projectleider kwetsbare ouderen<br>Zorggroep DOH<br>Tilburgseweg-West 100, 5652 NP Eindhoven<br>e-mail: C.devries@zorggroepdoh.nl

	Dr. G. Buunk, internist acute geneeskunde, Amphia<br>Molengracht 21, 4818 CK Breda<br>e-mail: gbuunk@amphia.nl<br><br>Dr. C.J.P.W. Keijsers, klinisch geriater<br>Jeroen Bosch Ziekenhuis<br>Henri Dunantstraat 1, 5223 GZ ’s-Hertogenbosch<br>e-mail: k.keijsers@jbz.nl<br><br>J. de Laat, unitmanager SEH<br>Jeroen Bosch Ziekenhuis<br>Henri Dunantstraat 1, 5223 GZ ’s-Hertogenbosch<br>e-mail: J.d.Laat@jbz.nl<br><br>Drs. M. Hermans, specialist ouderengeneeskunde<br>De Wever<br>Dr. Deelenlaan 6, 5042 AD Tilburg<br>e-mail: margriet.hermans@dewever.nl<br><br>Dr. M.A.C. de Jongh, klinisch epidemioloog<br>Netwerk Acute Zorg Brabant<br>Hilvarenbeekseweg 60, 5022 GC Tilburg<br>e-mail: m.d.jongh@nazb.nl<br><br>E.T.J. du Cloo, MSc, adviseur acute zorg<br>Netwerk Acute Zorg Brabant<br>Hilvarenbeekseweg 60, 5022 GC Tilburg<br>e-mail: e.ducloo@nazb.nl<br><br>Dr. M.A.C. Jansen, klinisch epidemioloog<br>Netwerk Acute Zorg Brabant<br>Hilvarenbeekseweg 60, 5022 GC Tilburg<br>e-mail: n.jansen@nazb.nl<br><br>Mw. M. van Daal, Bsc<br>Department of Internal Medicine<br>Maxima Medisch Centrum<br>De Run 4600, 5504 DB Veldhoven<br>e-mail: m.vandaal@maastricht.university.nl<br><br>Mw. S. van Berkel, registratiemedewerker<br>Netwerk Acute Zorg Brabant<br>Hilvarenbeekseweg 60, 5022 GC Tilburg<br>e-mail: s.vanberkel@nazb.nl<br><br>Mw. A. Hermans, registratiemedewerker<br>Netwerk Acute Zorg Brabant<br>Hilvarenbeekseweg 60, 5022 GC Tilburg<br>e-mail: a.lendering@nazb.nl<br><br>B. van Kuijk, registratiemedewerker

	Netwerk Acute Zorg Brabant<br>Hilvarenbeekseweg 60, 5022 GC Tilburg<br>e-mail: b.vankuijk@etz.nl<br><br>M. de Wilde, registratiemedewerker<br>Netwerk Acute Zorg Brabant<br>Hilvarenbeekseweg 60, 5022 GC Tilburg<br>e-mail: m.d.wilde@nazb.nl
**Sponsor**	Maxima Medisch Centrum<br>De Run 4600, 5504 DB Veldhoven
**Subsidising party**	Netwerk Acute Zog Brabant (NAZB)
**Independent expert (s)**	

Index

1. SUMMARY 6
2. INTRODUCTION AND RATIONALE 7
3. OBJECTIVES 7
4. METHODS 8
5. STUDY POPULATION 9
5.1.1 Population 9
5.1.2 Inclusion criteria 9
5.1.3 Exclusion criteria 10
5.1.4 Control group 10
5.1.5 Sample size 11
5.2 STUDY PROCEDURES 11
The APOP-screening 12
5.3 A SUMMARY OF THE NSC CARE PATHWAY 13
5.3.1 Control group 15
5.4 STUDY PARAMETERS / ENDPOINTS 16
5.4.1 Main study parameter/endpoint 16
5.4.2 Secondary study parameters/endpoints 16
5.5 STATISTICAL ANALYSIS 16
6. ETHICAL CONSIDERATIONS 17
6.1 Regulation statement 17
6.2 Recruitment and consent 17
6.3 Objection by minors or incapacitated subjects 17
6.4 Benefits and risks assessment, group relatedness 17
6.5 Compensation for injury 17
7. ADMINISTRATIVE ASPECTS, MONITORING AND PUBLICATION 18
7.1 Handling and storage of data and documents 18
7.2 Amendments 18
7.3 Public disclosure and publication policy 18
8. REFERENCES and FURTHER READING 19
Appendix 1 Voorstel Acute Geriatric Assessment (AGA) 20
Appendix 2 Questionnaire patient satisfaction 21

1. SUMMARY

Rationale: Approximately, 10-20% of older adult patients present with non-specific complaints (NSCs) at the emergency department (ED). NSCs are known as poorly described symptoms, such as ‘weakness’ and ‘fatigue’, often leading to an extensive differential diagnosis. Almost half of patients presenting with NSCs suffer from a serious underlying illness. Currently, a management protocol for patients with NSCs does not exist. Patients with NSCs are often under triaged, stay longer at the ED (ED-LOS) or hospital (HOSP-LOS) and are at a higher risk for complications during hospitalisation. A special care pathway for patients with NSCs was designed to resolve some of these problems and improve the efficiency of care at the ED.

Objective: To implement and evaluate a care pathway for older adults presenting with non-specific complaints at the emergency department.

Study design: A longitudinal multi-centre cohort with a stepped-wedge cluster design.

Study population: Older adults ≥70 years of age presenting with NSCs at the practice of the general practitioner (GP), the elderly care physician at a nursing home or the emergency department of the hospital will be evaluated for inclusion. Data from control patients will be collected retrospectively.

Recruitment: Recruitment will take place during workdays between Monday – Friday from 11:00 am – 20:00 pm and comprise of a study period of 6 months, according to the study protocol. The primary health care provider (such as a GP or elderly care physician at a nursing home) will indicate whether a patient is eligible for access to the care pathway and inform the specialist at the ED. Every referring care professional will have a card, which provides information on how to refer a patient to the care pathway and to whom. Patients can also enter the care pathway after triage at the ED, if the main complaint is non-specific. If the ED specialist registers access to the care pathway and the patient gives consent, the patient will be included and baseline data collected.

Intervention: If feasible, an ED-coach (passive or active form) will be appointed to each participant of the care pathway. The NSC will be evaluated in-depth, the patient will undergo APOP-screening during triage and if indicated, a comprehensive geriatric assessment will be performed after discharge from the ED at another department. The patient will be seen by a specialist or experienced resident in training at the ED, who will order a standard set of diagnostic tests and review the results. The APOP-risk score will guide further actions in the care pathway. A verification of the medication list will be performed <24 hours and a review of the medication will follow during admission.

Control cohort: Recruitment of controls will occur before implementation of the care pathway and on workdays between Monday – Friday from 11:00 am – 20:00 pm. The study period is estimated at approximately 6 months, according to study protocol. Each participating hospital will inform participants regarding their policy on data collection prior to implementing the care pathway. Data for controls within hospitals with access to CTcue will be collected retrospectively. The other participating hospitals will include eligible patients prospectively for the same estimated study period. After 30 days, the research nurse will evaluate complications that occurred after the patient leaves the care pathway.

Main study parameters/endpoints: Main endpoints are to evaluate the length of stay at the ED (ED-LOS) and exploring patient satisfaction on 4 established domains. Secondary objectives are evaluating the length of stay at the hospital (HOSP-LOS), discharge destination, medical diagnosis (at admission versus discharge), frequency of readmissions / revisits, 30-day mortality, loss of functional

status and costs-effectiveness of the care pathway. Study parameters are age, gender, main non-specific complaint, main diagnosis at ED-arrival and discharge, way of arrival (per ambulance, public transportation, etc.), main domain of NSC (somatic, nutrition, psychosocial, functional, mobility, falls), living situation (independent, care at home), diagnostic tests, specialist seeing the patient and consulting specialists and ED-logistics (time of arrival at ED, duration of triage, triage colour).

Nature and extent of the burden and risks associated with participation, benefit and group relatedness:
Participation in the care pathway is non-invasive. It includes specialized care for older adult patients presenting with NSCs at the emergency department. The burden regarding participation in this care pathway can be considered minimal

2. INTRODUCTION AND RATIONALE

Older adults, often frail, frequently present with poorly-defined symptoms leading to an extensive differential diagnosis [1]. These so called 'non-specific' complaints (NSCs), such as: 'feeling unwell', 'feeling fatigued' or 'feeling dizzy', are expressions of an acute medical problem in 50% of the cases [2]. Health care providers are challenged in the care of older adults with NSCs, due to their comorbidities, deteriorating cognition and the subjective nature of their complaints [3]. Therefore, a care pathway delivering special care for the older adult with NSC and concurrently providing an overview of the health status of a patient, might add value. Data from this care pathway can be used to guide a management protocol for elderly patients with NSCs at the emergency department (ED) to improve the quality of care.

Studies have shown that patients with NSCs have different characteristics compared to patients with specific complaints (SCs) [4]. Patients with NSCs have more comorbidities, use more medications, are at a higher risk of functional decline due to longer hospitalization and have a higher 30-day mortality rate compared to patients with SCs. Undertriage and unnecessary testing often contribute to a prolonged ED stay and frequent readmissions [5]. Furthermore, clinical experience of the health care provider seeing the patient with NSCs impacts diagnostic accuracy [6]. We aim to implement an integrated care pathway in different emergency departments of the Brabant area, focused on streamlining the care of patients with NSCs at the ED. This will be accomplished by evaluating the NSC in-depth (primary care), performing APOP (triage at the ED), screening certain domains of the CGA if a patient is high-risk according to APOP (somatic, nutrition, psychosocial, functional, mobility, falls), performing a standard set of diagnostic tests (at least: ECG, lab, urinalysis, X-thorax and bladder scan) and providing 'specialized care', as described in the methods section. In this care pathway, we will guide actions according to the APOP-risk score. After admission, a verification and review of the medication list will be performed. We hypothesize that the care pathway will primarily improve patient satisfaction and reduce the ED-length of stay. Reduced hospital length of stay, decreased 30-day mortality, improved functional status (daily activities, mobility, cognition), a reduction of (re-)admission rates for older adults with NSCs and reduced costs of care for this patient population might follow as a result of implementing this care pathway.

3. OBJECTIVES

1. The **primary objectives** of the care pathway are to evaluate:
- Length of stay at the ED (ED-LOS)
- Patient satisfaction on 4 domains

1. relief of symptoms (degree of relief and symptoms, duration until symptom relief, impact on function)
2. understanding the diagnosis and cause of symptoms, understanding prognosis
3. presence and understanding of the diagnostic, therapeutic and follow-up plan
4. reassurance during ED-stay

2. The **secondary objectives** of the care pathway are to evaluate:
- Length of stay at the hospital (HOSP-LOS)
- Discharge destination
- Medical diagnosis (at admission versus discharge)
- Frequency of readmissions / revisits
- 30-day mortality
- Loss of functional status
- Costs-effectiveness of the care pathway

4. METHODS

STUDY DESIGN
The following organizations will implement the care pathway:
- Catharina Ziekenhuis, Eindhoven, the Netherlands
- ETZ (Elisabeth-TweeSteden Ziekenhuis), Tilburg, the Netherlands
- Jeroen Bosch Ziekenhuis, ’s Hertogenbosch, the Netherlands
- Maxima Medisch Centrum, Veldhoven, the Netherlands
- St. Anna ziekenhuis, Geldrop, the Netherlands
- General practitioners of the Brabant area
- Nursing homes

Optional hospitals:
- Amphia, Breda, the Netherlands

Inclusion will take place during approximately 6 months after initiation of the study on workdays between Monday – Friday from 11:00 am – 20:00 pm.

The first hospitals were planned to initiate the study and start implementation of the care pathway and data collection in December 2019 or at the earliest possibility after approval of the study protocol. Due to the COVID-pandemic and its burden on hospital-care throughout the Netherlands, implementation could not take place as planned. The Jeroen Bosch Hospital had started the care-pathway, but had to discontinue after a few weeks due to the massive burden of COVID-care.

Considering that the COVID-pandemic, which resulted in a drop of regular care, may have had an influence on the processes in the ED and the number of elderly people presenting with non-specific complaints, it was decided to temporarily withhold the inclusion of control patients and implementation of the care-pathway. The estimation was made that the COVID-pandemic would be a great confounder in results when comparing a pre-pandemic and post-pandemic population. Therefore it was decided to start over with inclusion of the control-group. In July and August 2020 the participating hospitals were consulted and asked if they had resumed their usual care and how their ED and population had changed. All hospitals agreed to restart inclusion of the control-group form August 2020 onward. Since the numbers of elderly people with non-specific complaints are

expected to be lower than before the COVID-pandemic, it is possible that the period for inclusion for the control group has to be extended. The planned start for implementation of the care-pathway is January 2021, dependent on inclusion of the control-group and the evolution of the COVID-pandemic.

At this moment, October 2020, we are experiencing a second wave of the COVID-pandemic throughout the Netherlands. During the first outbreak, the hospitals in Noord-Brabant especially were in state of crisis, with a drop in the rates of regular care. With this impact in mind, we decided to withhold the care-pathway and include a post-COVID control-group. However, the global opinion is becoming that COVID is here to stay, and it will become part of the day to day business both at social and hospital-level. Therefore the aim of hospitals throughout the Netherlands is to arrange the COVID care in the best possible manner, but also assuring regular care to continue as much as possible. With this in mind, the research team decided not to further postpone the inclusion of the control group and planned implementation of the care-pathway is estimated at January 2021. We are aware of the changes in organisation of care that come with the periodical higher burden of COVID-care, so we plan to monitor changes in organisation at the Emergency Department during the inclusion period in each participating hospital.

5. STUDY POPULATION

5.1.1 Population

Older adult patients presenting with a non-specific main complaint at the practice of their general practitioner (GP) will be screened for inclusion. If a patient meets the inclusion criteria, the GP will refer the patient to a hospital that implemented the care pathway and notify the specialist at the ED (internist, ED-doctor or elderly care physician). Every referring care professional will have a card, which provides information on how to refer a patient to the care pathway and to whom. Patients not referred by the GP can enter the care pathway in several other ways:
- if referred by the practitioner at a nursing home
- if triage at the ED points to a NSC

5.1.2 Inclusion criteria

In order to be eligible to participate in this study, a subject must meet all of the following criteria:

- Indicated for admission to hospital, ***and***
- Age ≥ 70 years, ***and***
- A non-specific main complaint at presentation, such as:—
  1. <u>somatic problems</u>:
     - *weakness:* physical weakness in the body limiting the patient to perform daily activities
     - *not feeling well:* patients expressing a passive behaviour due to not feeling well physically or mentally
     - *change in nutritional status:* an abrupt decline of eating and/or drinking, compared to previous eating habits
     - *unexplained weight loss:* an ongoing weight loss or recent weight loss of more than 10% of baseline in the previous month, not related to a modified diet or exercise
  2. <u>a higher demand of care:</u>

- *loss of independency:* an abrupt or ongoing decline of being able to perform daily activities independently
- *a necessity for a change in the living situation*, due to a higher demand of care
- *a necessity for 24-7 care*, not indicated previously

3. cognitive problems:
- *disorientation:* inability to recall current date, name or current environment
- *changes in behaviour:* unexplained agitation, abrupt changes in behaviour
- *cognitive decline:* abrupt decline in cognitive performances

4. functional status:
- *loss of mobility:* change in functional status leading to limited mobility

5. unexplained falls: a fall not related to extrinsic factors such as poor lighting, unsafe stairways, and irregular floor surfaces or to a precise medical or drug-induced cause

5.1.3 Exclusion criteria

A potential subject who meets any of the following criteria will be excluded from participation in this study:

- Specific (main) complaint coupled to a diagnosis (pain, dyspnea, cough, localised weakness, swollen extremity, diarrhea, dysuria, bleeding, syncope, skin lesions, vertigo, palpitations, e.g.)
- Age <70 years
- Patient refusing data collection or participation in the care pathway

5.1.4 Control group

For patients included in the study as controls, the same inclusion and exclusion criteria apply. Controls will not be specifically asked for informed consent, as each participating hospital will sign a form to collect data for control patients prior to implementing the care pathway. Additionally, it is the responsibility of each participating institution to inform their patients on the policies regarding data collection in study settings.
Obtaining the retrospective control-group will be done by CTcue, if possible. CTcue is a search engine in which unstructured data from an Electronic Health Record (EHR) can be easily found by adding specific queries in to the search engine. These data will be retrieved anonymously according to our defined in- and exclusion criteria, study parameters and endpoints. Specialists at the ED will be unaware of the patients functioning as controls during this study, since data for controls will be collected retrospectively. After 30 and 90 days, the research nurse will evaluate complications that occurred after the patient leaves the care pathway.

In the participating hospitals without access to CTcue, control patients will be gathered prospectively. The ED specialist will save the patient-number of eligible patients in the care pathway (before it is implemented). The specialist in charge of the patient is allowed to use patient data for research, without consent when data is anonymised. The specialist may grant this right to a third party, in this case the research team. This exchange of data has to be approved by the involved departments, which will be the department of Internal Medicine, Geriatrics and the Emergency Department.

5.1.5 Sample size

We hypothesize that implementing the care pathway will reduce the mean ED-LOS with 10%. In a prospective study about the quality of Acute Care, by M.N.T. Kremers (submitted for publication), the mean length of stay at the ED in the Maxima Medical Centre is three hours and twenty minutes (95% CI 3:00-3:40, SD=1.17). Assuming a normal distribution, a two sided test, power of 80% and a significance level (a) of 0.05, a minimum of 466 patients need to be included, 233 patients in both control and intervention group. We aim to include 300 patients in each group, with a estimated timeframe of one year. Each hospital will gather their own control-group before implementation of the care-pathway. A minimum number of control-patients will be established for each participating hospital, dependent of number of annual patient-visits at the Emergency Department.

5.2 STUDY PROCEDURES

The primary health care provider or GP will be the first to screen older adult patients presenting with a non-specific main complaint at their practice and will evaluate whether the patient is eligible for inclusion. Patients not referred by the GP can enter the care pathway if referred by the elderly care physician at a nursing home or if triage at the ED points to a NSC. If a patient meets all inclusion criteria, the primary health care provider will notify the specialist at the ED. Every referring care professional will have a card, which provides information on how to refer a patient to the care pathway and to whom. If possible, an ED-coach will be assigned to each participant of the care pathway, who meets the urgency criteria. The patient will be seen by an experienced doctor, according to hospital policy.

A schematic overview:

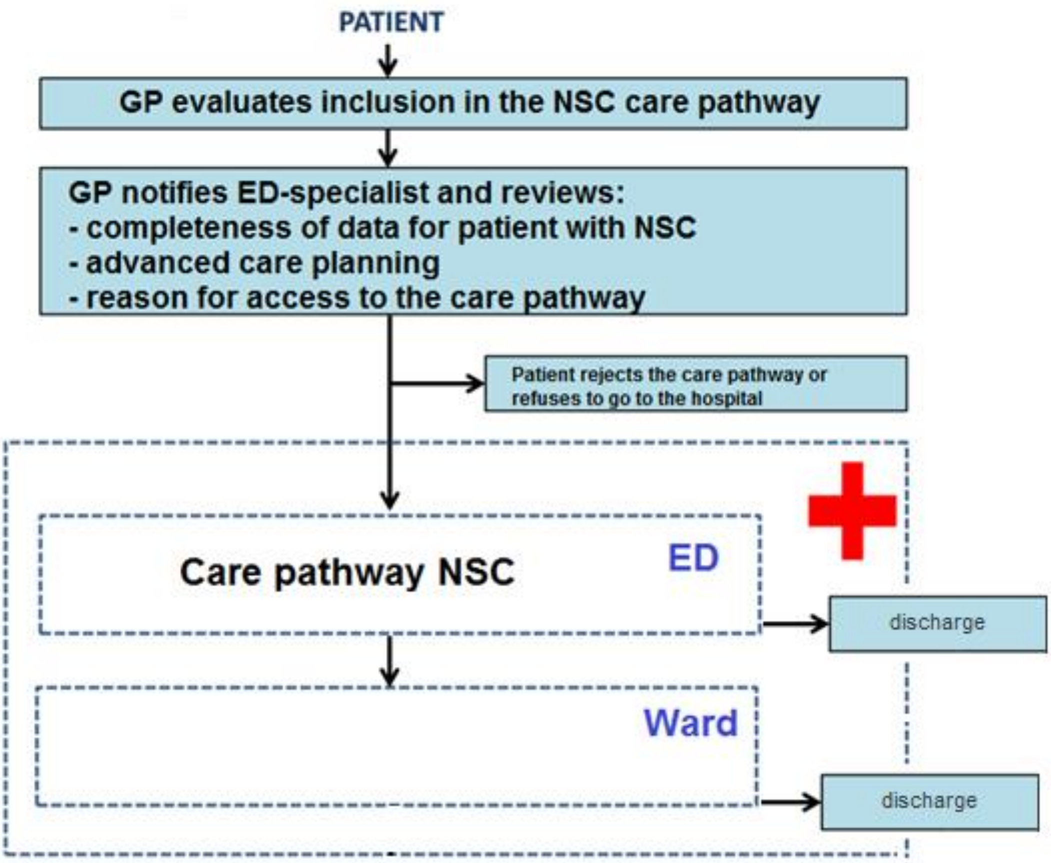

The care pathway will comprise of special attention and focused care for the patient with NSC at the ED (see paragraph 4.4)

The APOP-screening

Each triage of the participating hospital will be trained for the APOP-screening.
The APOP-screener [11] consists of 9 questions:
Patient information, filled in by the nurse
1. Age (≥ 70 year)
2. Gender
3. Did the patient arrive per ambulance?
Questions for the patient
4. Did you need help from someone? (e.g. preparing meals, housekeeping)
5. Did you need help with showering or bathing?
6. Have you been admitted to the hospital in the previous 6 months?
7. Have you been diagnosed with dementia?
    - Yes, screening ended
    - No, ask the following questions
- Can you reproduce the current year?
- Can you reproduce months of the year in the reverse order?

These questions lead to a risk of functional decline, cognitive decline or both, according to the APOP-screener. Four scenarios are:

1. <u>Low risk</u>, no supplementary actions recommended

2. High risk of functional decline - the following actions are recommended among others; in case of suspected dehydration at presentation: starting early fluid administration, in case of admission: limiting the length of stay at the ED and organizing early transfer to the ward.
3. High risk of cognitive decline - the following actions are recommended among others; Making sure the patient has a comfortable bed, has adequate lightning in the room and knows how to call for help.
4. High risk of functional and cognitive decline - the following actions are recommended among others; consider the diagnosis delirium and an internist geriatric medicine or clinical geriatrician can be consulted for a comprehensive geriatric assessment after discharge from ED.

Patient satisfaction (Appendix 2)
Patient satisfaction will be evaluated for each participant of the care pathway, directly after the patient leaves the care pathway. A preliminary analysis from a recently performed study by Kremers (MD) et al. showed some common themes for patients presenting to the ED. These main domains include:

1. relief of symptoms (degree of relief and symptoms, duration until symptom relief, impact on function)
2. understanding the diagnosis and cause of symptoms, understanding prognosis
3. presence and understanding of the diagnostic, therapeutic and follow-up plan
4. reassurance

These domains will be evaluated for patients in the care pathway as well.

5.3 A SUMMARY OF THE NSC CARE PATHWAY

1. Patients will be included in the care pathway by a general practitioner, elderly care physician or triagist at the ED. Their tasks include:
   - Check of inclusion and exclusion criteria
     - guidelines of the study will be available on a card and in a digital format
   - Including patients via healthcare system or after contacting the specialist at the ED (internist, ED-doctor or elderly care physician)
   - Digital registration of a participant in the care pathway

2. The triagist or a trained nurse will perform the APOP, using the APOP-app on a tablet or in the electronic medical records. If possible, the ED-coordinator will assign an ED-coach to a participant of the study.

3. Every hospital will have their own system coupled to the Care Domain of the primary care provider and every participant will consent for sharing information on their frailty status. Patients will be seen by a doctor and a nurse. Three key players in this care pathway are:
   - I – Nurse
   - II – Specialist at the ED (specialist or resident in training):
     a. in case of non-trauma patients: internist, emergency physician or elderly care physician
     b. In case of trauma-patients: surgeon, orthopedist or emergency physician
     c. preferably/ if possible no interns or untrained residents will see the patient with NSCIII
     d. reviewing Frailty information from sources (primary care physician, informal caregiver, own findings, APOP; see also Appendix 1)

- ED-coach (if possible)
  a. this can either be a secretary, nurse or a doctor
  b. there is either no ED-coach, passive ED-coach or an active ED-coach

4. Tasks of the ED-coach (no ED coach, passive ED-coach or active ED-coach):
   - It is possible that no ED-coach can be appointed.

   If an ED-coach can be appointed however, they can either be active or passive in patient care and perform the following tasks.
   If a passive ED-coach is feasible:
   - Checking progress at the ED for the participant every 30 minutes

   If an active ED-coach is feasible:
   - Coordinating care and communicating with the doctor and nurse taking care of the participant.
   - Checking completeness of data for inclusion, reviewing medical history, calling the local responsible for medication verification <24 hours after admission, evaluating care at home, assess patient satisfaction for participant of the care pathway
     - Checking progress at the ED for the participant every 30 minutes
     - Checking adherence to the recommended APOP-guidelines after completion

5. Tasks of the nurse:
   - Performing the APOP screening (potential)
   - Conventional care at the ED
   - Performing the ordered diagnostic tests
   - Performing actions following the APOP-risk score

6. Tasks of the doctor:
   - Conventional care at the ED
   - Ordering a standard set of diagnostics, at least: ECG, lab, urinalysis, X-thorax and bladder scan.

7. Actions following APOP-screening:
   1. Low risk, no supplementary actions recommended
   2. High risk of functional decline - the following actions are recommended among others; in case of suspected dehydration at presentation: starting early fluid administration, in case of admission: limiting the length of stay at the ED and organizing early transfer to the ward.
   3. High risk of cognitive decline - the following actions are recommended among others; Making sure the patient has a comfortable bed, has adequate lightning in the room and knows how to call for help.
   4. High risk of functional and cognitive decline - the following actions are recommended among others; consider the diagnosis delirium and an internist geriatric medicine or clinical geriatrician can be consulted for a comprehensive geriatric assessment after discharge from ED.

8. Discharge from the ED:
- Possibilities include: admission to the ward, discharge to home, discharge to facility, discharge to short stay facility for primary care, discharge to geriatric rehabilitation.
- Each study participant will be followed-up after leaving the care pathway.
- A review of the medication list will be performed by the doctor at the ward, elderly care physicianthe clinical pharmacologist or hospital pharmacist

9. Tasks of the research nurse / researcher:

- Collecting data for the database (see study parameters and endpoints)
- Collecting data for each participant 30 days after leaving the care pathway

5.3.1 Control group

Specialists in the hospitals with CTcue will be unaware of the patients functioning as controls during this study, since data for controls will be collected retrospectively. In the participating hospitals without access to CTcue, control patients will be gathered prospectively. The aim is that each participating hospital gathers a control group which will be compared with their intervention group.

5.4 STUDY PARAMETERS / ENDPOINTS

5.4.1 Main study parameter/endpoint

Baseline characteristics:

- Age in years
- Gender (male/female)
- Referral category NSC
  - Somatic
  - Higher demand of care
  - Cognitive problems
  - Functional status
  - Unexplained falls

- Main diagnosis at ED (DOT/DBC/ICD10)
- Way of arrival (per ambulance, public transportation, own transport, etc.)
- APOP-score
- Adherence to the recommended actions after APOP-screening and risk of functional decline
- Specialist seeing the patient and consulting specialists during ED-stay
- Diagnostic tests (X-ray, EKG, urinalysis, bladder scan and the frailty lab)
- ED-logistics (time of arrival at ED, duration of triage, triage colour, ED loss etc)
- In the case of admission: duration of admission (days) and reason for admission
- Living situation (independent, care at home, etc)
- Zip (postal code)
- Discharge destination (home, facility, care at home)
- Change in medication list after review
- Patient satisfaction

Endpoints are:

- Diagnostic procedures before and after implementation of the care path (quantity)
- ED length of stay – ED-LOS (in hours)
- Hospital length of stay – HOSP-LOS (in days)
- Readmissions in 90 days (yes/no)
- 30-day mortality
- Revisits in 30 days (yes/no)
- Patient satisfaction

5.4.2 Secondary study parameters/endpoints

Economic perspectives (such as costs) of the care pathway will be evaluated.

5.5 STATISTICAL ANALYSIS

Primary and secondary study parameters and endpoints are described in 'Study parameters/endpoints'. All statistical analyses will be performed with SPSS 24.0 for Windows (IBS, SPSS Statistics, Chicago, IL, USA). Descriptive statistics will be used for frequencies, percentages, means and standard deviations. Continuous variables will be analysed using independent t-tests, whereas the categorical data will be analysed by chi-square or Fisher's exact test. Median and interquartile ranges of non-normally distributed categorical and metric variables of the study will be

assessed using Mann-Whitey-U-test. Prevalence of the most frequent diagnosis (coded ICD-10) at discharge will be presented in percentages. HR and corresponding 95% confidence intervals will be calculated. Generalized Estimating Equations (GEEs) will be used, which adjust for observations made within the same hospitals being more alike. All tests will be performed using a significance level of alpha =0.05. A two-sided p-value <0.05 will be considered significant.

6. ETHICAL CONSIDERATIONS

6.1 Regulation statement

The study will be conducted according to the principles of the Declaration of Helsinki (version 2004, May 22, 2007; www.wma.net) in accordance with the Medical Research Involving Human Subjects Act (WMO).

6.2 Recruitment and consent

Older adults presenting to their primary health care provider with a NSC will be considered for the care pathway. Every referring care professional will have a card, which provides information on how to refer a patient to the care pathway and to whom. Patients not referred by the GP can enter the care pathway if referred by the practitioner at a nursing home or if triage at the ED points to a NSC. If a patient meets all inclusion criteria, the primary health care provider will notify the specialist at the ED (internist, emergency physician or elderly care physician). If eligible and feasible, an ED-coach will introduce the patient to the care pathway and make sure the patient receives special care. Each participating hospital for the care pathway will sign a form to collect data for control patients beforehand.

6.3 Objection by minors or incapacitated subjects

Minors will not be included in this study. For incapacitated subjects, the legal representative will be encountered for informed consent.

6.4 Benefits and risks assessment, group relatedness

Participation in the care pathway is non-invasive. It includes specialized care for older adult patients presenting with NSCs at the emergency department. The burden regarding participation in this care pathway is comparable for capacitated adults and can be considered minimal.

6.5 Compensation for injury

The sponsor/investigator has a liability insurance which is in accordance with article 7 of the WMO. Hospitals and caregivers / organisations other than hospitals have responsibilities and according insurance for the care of their patients.

7. ADMINISTRATIVE ASPECTS, MONITORING AND PUBLICATION

7.1 Handling and storage of data and documents

All data will be handled confidentially. Participant data will be safeguarded and coded by a subject identification code, therefore he or she will not be identifiable. Data will be handled according to the Dutch Personal Data Protection Act and stored in Research Manager. When it is necessary to be able to trace data for an individual subject, a subject identification code list can be used to link the data. They key to this list will only be given to the principal investigator. Only qualified research personnel will have access to confidential data. Research data will be stored for at least 15 years.

7.2 Amendments

Amendments are changes made to the research after a favourable opinion by the accredited METC has been given. All amendments will be notified to the METC that gave a favourable opinion.

A ‘substantial amendment’ is defined as an amendment to the terms of the METC application, or to the protocol or any other supporting documentation, that is likely to affect to a significant degree:

- the safety or physical or mental integrity of the subjects of the study;
- the scientific value of the study;
- the conduct or management of the study;
- the quality or safety of any intervention used in the study.

All substantial amendments will be notified to the METC and to the competent authority.

Non-substantial amendments will not be notified to the accredited METC and the competent authority, but will be recorded and filed by the sponsor.

7.3 Public disclosure and publication policy

All results produced from this study will be published after agreements with participating institutions and involved researchers. No identifiable patient information will be published.

8. REFERENCES and FURTHER READING

1. Karakoumis J, Nickel CH, Kirsch M, Rohacek M, Geigy N, Muller B, et al. Emergency Presentations With Nonspecific Complaints-the Burden of Morbidity and the Spectrum of Underlying Disease: Nonspecific Complaints and Underlying Disease. Medicine (Baltimore). 2015;94(26):e840.

2. Rutschmann OT, Chevalley T, Zumwald C, Luthy C, Vermeulen B, Sarasin FP. Pitfalls in the emergency department triage of frail elderly patients without specific complaints. Swiss Med Wkly. 2005;135(9-10):145-50.

3. Murray AM, Toussaint A, Althaus A, Lowe B. The challenge of diagnosing non-specific, functional, and somatoform disorders: A systematic review of barriers to diagnosis in primary care. J Psychosom Res. 2016;80:1-10.

4. Wachelder JJH, Stassen PM, Hubens L, Brouns SHA, Lambooij SLE, Dieleman JP, et al. Elderly emergency patients presenting with non-specific complaints: Characteristics and outcomes. PLoS One. 2017;12(11):e0188954.

5. Aminzadeh F, Dalziel WB. Older adults in the emergency department: a systematic review of patterns of use, adverse outcomes, and effectiveness of interventions. Ann Emerg Med. 2002;39(3):238-47.

6. Nemec M, Koller MT, Nickel CH, Maile S, Winterhalder C, Karrer C, et al. Patients presenting to the emergency department with non-specific complaints: the Basel Non-specific Complaints (BANC) study. Academic emergency medicine. 2010;17(3):284-92.

7. Mahler SA, Riley RF, Hiestand BC, Russell GB, Hoekstra JW, Lefebvre CW, et al. The HEART Pathway randomized trial: identifying emergency department patients with acute chest pain for early discharge. Circ Cardiovasc Qual Outcomes. 2015;8(2):195-203.

8. Van Herck P, Vanhaecht K, Sermeus W. Effects of clinical pathways: do they work? Journal of Integrated Care Pathways. 2004;8(3):95-105.

9. Hertwig R, Meier N, Nickel C, Zimmermann P-C, Ackermann S, Woike JK, et al. Correlates of diagnostic accuracy in patients with nonspecific complaints. Medical Decision Making. 2013;33(4):533-43.

10. Conroy SP, Ansari K, Williams M, Laithwaite E, Teasdale B, Dawson J, et al. A controlled evaluation of comprehensive geriatric assessment in the emergency department: the 'Emergency Frailty Unit'. Age and ageing. 2013;43(1):109-14.

11. de Gelder J, Lucke JA, Blomaard LC, Booijen AM, Fogteloo AJ, Anten S, et al. Optimization of the APOP screener to predict functional decline or mortality in older emergency department patients: Cross-validation in four prospective cohorts. Exp Gerontol. 2018;110:253-9.

12. Agarwal K., et al. Failure to thrive in elderly adults: Evaluation. UptoDate Online, last updated: May 02, 2018

Appendix 1 Acute Geriatric Assessment (AGA) in combination with history taking and routine physical examination

	Geschatte Tijdsinvestering "intern onderzoek" in min	Geschatte Extra tijdsinvestering AGA in min	Door wie (lokaal af te spreken)<br><br>Coach: C<br>Arts: A<br>Apotheek: Ap<br>Verpleegkundige: V
**Voorbereidende fase (6 min)**			
**Voorgeschiedenis (**uitzoeken)	3	-	c/a
**Medicatieoverzicht** (uitzoeken; lokaal borgen medicatie verificatie)	2		c/ap
**Is er wilsverklaring**/ACP		1	c/a
**Onderzoekende fase (22 minuten)**			
**Reden van insturen**	0.5		c/a
**Anamnese:**			
+ speciele anamnese	3		a
Tractus anamnese:	3		a
+ zijn er geheugenproblemen, delier of stemmingsproblemen		1	a
+ problemen met eten/gewicht		0.5	a
**Heteroanamnese**			
Aanvulling/discrepanties op bovenstaande vragen		3	a
**Functionele status:**			
+ wat kan pt nu nog, wat kon pt 14 dgn geleden		1	c/v
+ hoe is koken/boodschappen geregeld		1	c/v
+ actieradius (lopen met of zonder hulpmiddel, fietsen, autorijden)		1	c/v
**Sociale status:**			
+ hoe woont patiënt en volstaat dat nu (nu, -14dgn)? Mantelzorg? Prof zorg?		1	c/v
**Lichamelijk onderzoek**			
+ Algemeen intern	5		a
+ zelfstandig tot lopen (bij nee-> neurologisch onderzoek uitbreiden)		2	a
+ eerste indruk psyche + orientatie trias		0.5	a
**Concluderende fase (10 min)**			
**Aanvullend onderzoek:**			
+ ger lab (geen vitamine)/ urine ao/bladder/ECG	0	0	v
**Maken behandelproblemen** en beleid in 4 assen	4	2	a
**bespreken beleid** incl reanimatiebeleid en verwachtingen pt/familie	4		a
Geschatte duur interne pt in kaart brengen incl bespreken beleid met pt	*24.5 min*<br>*19 min arts*<br>*5.5 min coach/apotheker*		
Geschatte extra duur AGA		*+14 min*<br>*9 min arts*<br>*5 min VPK/coach*	

Appendix 2 Questionnaire patient satisfaction

Zorgpad ouderen met niet-specifieke klachten op de Spoedeisende Hulp

-Vragenlijst-

U bent behandeld op de Spoed Eisende Hulp. Wij willen graag weten hoe u de zorg ervaren heeft en of u goed geholpen bent op de Spoedeisende Hulp.

Wilt u onderstaande vragen beantwoorden door het meest passende cijfer te omcirkelen en bij vraag 3 het meest passende vakje aan te kruisen?

	**Geen last**										**Zeer veel last**
1. Hoeveel last had u van uw klachten bij binnenkomst op de Spoedeisende Hulp?	0	1	2	3	4	5	6	7	8	9	10
2. Hoeveel last had u van uw klachten bij het verlaten van de Spoedeisende Hulp?	0	1	2	3	4	5	6	7	8	9	10

3. Zijn uw klachten behandeld?

- ☐ Ja, en de behandeling heeft voldoende geholpen
- ☐ Ja, maar de behandeling heeft niet voldoende geholpen
- ☐ Ja, en ik word nog steeds behandeld
- ☐ Nee, maar ik had wel graag een behandeling willen hebben
- ☐ Nee, maar ik wilde ook geen behandeling voor mijn klacht(en)

	**Helemaal niet**	**Vrijwel niet**	**Matig**	**Redelijk**	**Goed**	**Volledig**
4. Begreep u de uitleg op de Spoedeisende Hulp over de oorzaak van uw klachten?	1	2	3	4	5	6

☐ Ik heb geen uitleg over de oorzaak van mijn klachten gekregen

	**Helemaal niet**	**Vrijwel niet**	**Matig**	**Redelijk**	**Goed**	**Volledig**
5. Begrijpt u waarom de onderzoeken en/of behandelingen op de Spoedeisende Hulp uitgevoerd zijn? (bijv. bloed prikken, infuus)	1	2	3	4	5	6

	**Helemaal niet**	**Vrijwel niet**	**Matig**	**Behoorlijk**	**Goed**	**Volledig**
6. Begrijpt u wat er nog voor u (of uw klachten) gedaan moet worden tijdens opname in het ziekenhuis of thuis?	1	2	3	4	5	6
7. Voelde u zich gerustgesteld na uw bezoek van de Spoedeisende Hulp?	1	2	3	4	5	6
8. Denkt u dat u en/of uw klachten uitgebreider onderzocht hadden moeten worden?	1	2	3	4	5	6
9. Bent u tevreden over de totale duur van uw verblijf op de Spoedeisende Hulp?	1	2	3	4	5	6
10. Voelde u zich veilig tijdens uw verblijf op de Spoedeisende Hulp?	1	2	3	4	5	6
11. Werd er door de zorgverleners naar u geluisterd tijdens uw verblijf op de Spoedeisende Hulp?	1	2	3	4	5	6
12. Had u vertrouwen in de deskundigheid van de zorgverleners op de Spoedeisende Hulp?	1	2	3	4	5	6
	**Zeer slecht**					**Zeer goed**

13. Welk cijfer zou u de Spoedeisende Hulp geven op een schaal van 0-10?	0	1	2	3	4	5	6	7	8	9	10
