# Supplementary material for: Implementation of a care-pathway at the emergency department for older people presenting with nonspecific complaints; a protocol for a multicenter parallel cohort study
Source: PLoS One. 2023 Aug 29;18(8):e0290733. doi: 10.1371/journal.pone.0290733 (PMC10464958; doi:10.1371/journal.pone.0290733)
Supplement: Attachment — Submitted filename: renamed_9141c.docx [file pone.0290733.s004.docx]

# COMMENTS – Manuscript PONE-D-23-17637

Title: “Implementation of a care-pathway at the emergency department for older people presenting with nonspecific complaints; A protocol for a multicenter stepped wedged cohort study”

**Important note: This review pertains only to ‘statistical aspects’ of the study and so ‘clinical aspects’ [like medical importance, relevance of the study, ‘clinical significance and implication(s)’ of the whole study, etc.] are to be evaluated [should be assessed] separately/independently. Further please note that any ‘statistical review’ is generally done under the assumption that (such) study specific methodological [as well as execution] issues are perfectly taken care of by the investigator(s). This review is not an exception to that and so does not cover clinical aspects {however, seldom comments are made only if those issues are intimately / scientifically related & intermingle with ‘statistical aspects’ of the study}. Agreed that ‘statistical methods’ are used as just tools here, however, they are vital part of methodology [and so should be given due importance]. I look at the manuscript in/with statistical view point, other reviewer(s) look(s) at it with different angle so that in totality the review is very comprehensive. However, there should be efforts from authors side to improve (may be by taking clues from reviewer’s comments). Therefore, please do not limit the revision only (with respect) to comments made here.**

**COMMENTS:** Although this manuscript is well drafted and the study seems to have enough potential (is planned for very important/useful topic), I have few observations/concerns (different opinion) which are little serious and are given below:

It is appreciated that the protocol suggests to use a novel research study design [The stepped wedge cluster randomised trial or cohort study which is increasingly being used in the evaluation of service delivery type interventions] with such mention in the title (A protocol for a multicenter stepped wedged cohort study), however, the actual design details are not described/given/clarified adequately. Even ‘Methods-Trial design’ section (lines 77-80, where details are expected) is very short and does not elaborate on this. It only says that “This study is a longitudinal multicenter cohort trial with a stepped wedged design. The study will be organized in hospitals in the Noord-Brabant area, the Netherlands, in which general practitioners (GPs), elderly care physicians (ECPs), Emergency Physicians (EPs), geriatricians and internists will collaborate. Participating hospitals can be general or teaching hospitals” which is not adequate. It is well recognised that design deserves as much consideration as analysis [This is pasted from one standard textbook on ‘Medical Research Methodology’] and I am sure that the authors already know this.

According to my information/knowledge, this design involves random and sequential crossover of clusters from control to intervention until all clusters are exposed. A classical article on this design in BMJ by Hemming [Hemming K, Haines T P, Chilton P J, Girling A J, Lilford R J. The stepped wedge cluster randomised trial: rationale, design, analysis, and reporting BMJ 2015; 350 :h391 doi:10.1136/bmj.h391] is unfortunately (seems to had not used &) not quoted in the manuscript. This article says “In a stepped wedge study, the sample size calculation is complicated by the need to allow for the confounding effect of calendar time, and this means that the standard **design effect** is no longer applicable. Compared with a simple parallel study, where no such confounding occurs, the time effect tends to degrade the precision of the study and increase the sample size needed to achieve adequate power”. Even standard ‘design effect’ seems to have not been applied {or if applied is not mentioned/clarified in ‘Statistical Analysis-Sample size’ section (lines 179-191)}. If because of (due to) “crossover” cluster ‘design effect’ is not applicable, that needs to clarify and quote the reference. In fact, there is a complete absence of any reference on this design or methodology {I could not identify any such reference out 18 mentioned/listed in this manuscript}. Is not it surprising?

Is article quoted as number 17 [Kremers MNT, Mols EEM, Simons YAE, van Kuijk SMJ, Holleman F, Nanayakkara PWB, et al. Quality of acute internal medicine: A patient-centered approach. Validation and usage of the Patient Reported Measure-acute care in the Netherlands. PLOS ONE. 2020;15(12):e0242603] used for required sample size estimation? Since the design used and focus of that study is entirely different {In this study, we primarily aim to assess the validity of the PRM-acute care in internal medicine patients and secondly, to gain insight into the current perceived quality of acute care, with the overarching goal to use the PRM-acute care in daily practice and improve patient-centered care in the ED}, I wonder, ‘how’ that helped? Will you please explain? [essential since it is quoted here]. Because the earlier quoted Hemming’s article on page 4 says “As yet, there is no specific adaptation of design effects, for calculating the power or sample size in a cohort stepped wedge trial, nor implementation in a statistical package for this design.”, I would like to learn (as Hemming’s article is of 2015 & you may be aware or might have used later/newer developments).

Except these minor points, the article is acceptable. Nevertheless, mind you that as pointed out in ‘important note’ above “This review pertains only to ‘statistical aspects’ of the study and so ‘clinical aspects’ should be assessed separately/independently. ‘Minor Revision’ is recommended.
